# Offering Outworld Experiences to In-Patients With Dementia Through Virtual Reality: Mixed Methods Study

**DOI:** 10.2196/45799

**Published:** 2023-08-31

**Authors:** Maria Matsangidou, Theodoros Solomou, Fotos Frangoudes, Ersi Papayianni, Constantinos S Pattichis

**Affiliations:** 1 CYENS Centre of Excellence, Nicosia, Cyprus; 2 Department of Computer Science, University of Cyprus, Nicosia, Cyprus; 3 Archangelos Michael Elderly People Nursing Home/Rehabilitation Centre for Patients with Alzheimer, Nicosia, Cyprus

**Keywords:** virtual reality, dementia, patient-centered design, psychophysiological responses, behavioral and psychological symptoms, in-patient, VR, symptom management, quality of life, intervention, mental health, mental disorder, dementia care

## Abstract

**Background:**

Research has suggested that institutionalization can increase the behavioral and psychological symptoms of dementia. To date, recent studies have reported a growing number of successful deployments of virtual reality for people with dementia to alleviate behavioral and psychological symptoms of dementia and improve quality of life. However, virtual reality has yet to be rigorously evaluated, since the findings are still in their infancy, with nonstatistically significant and inconclusive results.

**Objective:**

Unlike prior works, to overcome limitations in the current literature, our virtual reality system was co-designed with people with dementia and experts in dementia care and was evaluated with a larger population of patients with mild to severe cases of dementia.

**Methods:**

Working with 44 patients with dementia and 51 medical experts, we co-designed a virtual reality system to enhance the symptom management of in-patients with dementia residing in long-term care. We evaluated the system with 16 medical experts and 20 people with dementia.

**Results:**

This paper explains the screening process and analysis we used to identify which environments patients would like to receive as an intervention. We also present the system’s evaluation results by discussing their impact in depth. According to our findings, virtual reality contributes significantly to the reduction of behavioral and psychological symptoms of dementia, especially for aggressive, agitated, anxious, apathetic, depressive, and fearful behaviors.

**Conclusions:**

Ultimately, we hope that the results from this study will offer insight into how virtual reality technology can be designed, deployed, and used in dementia care.

## Introduction

Globally, there are estimated to be 55 million patients with dementia [1]; therefore, the World Health Organization set out a global action plan to improve these patients’ quality of life [2,3]. Dementia refers to a set of conditions that affect memory, thinking, and orientation and is often accompanied by behavioral and psychological symptoms (BPSDs), which are characterized by aggressive behaviors toward oneself and others, restlessness, irritability, depression, apathy, and lack of motivation [1,4-7].

The majority of interventions designed to prevent or reduce BPSDs in patients with dementia are based on pharmacological medications and physical barriers that are linked to adverse effects; the worsening of the patient’s condition; and increases in anxiety, distress, and aggressive behaviors [8-12]. Meanwhile, there is a growing body of evidence demonstrating positive outcomes from nonpharmacological interventions for people with dementia that do not cause any of the aforementioned adverse effects [13-16]. Among the most common practices is sensory stimulation (eg, aromatherapy, music, and massages), as well as exposure to interesting and alternative environments, art, and reminiscence therapy. Therefore, in accordance with the World Health Organization’s global action plan [3], best practices should reflect the use of pharmacological interventions and physical restraints only when nonpharmacological interventions have failed to be effective in treating complex cases.

The use of computer technology and, in particular, virtual reality (VR) has enabled the use of nonpharmacological interventions, as a result of their ability to immerse the person with dementia in interesting and alternative environments that offer feedback based on multisensory stimulations [17,18]. According to recent studies, VR can serve as a viable and acceptable method for enhancing the engagement and enjoyment of patients with dementia [18,19]. A number of recent studies have also explored the effectiveness of VR in alleviating BPSDs and improving the quality of life of patients with dementia, with inconclusive results [11,20-23]. Specifically, some results have suggested a reduction in agitated, apathetic, and depressive responses but no improvements in cognitive function [20-22], while others have demonstrated that when VR is administered, it can preserve cognitive function [21].

Building on the above findings, to examine if VR can play a fundamental role in the reduction of BPSDs and the enhancement of quality of life for patients with dementia, we co-designed a VR system, reflecting on comments from 44 patients with dementia and 51 medical experts, to improve the symptom management of patients with dementia residing in long-term care services. We then evaluated the effectiveness of the system with 20 patients with dementia.

## Methods

### Ethics Approval

Patients diagnosed with dementia were recruited from a national Alzheimer disease and dementia hospital, in which they were residing and receiving care. Ethical approval was obtained from the National Bioethics Committee (approval number: Eebk ep 2022 56). All participants signed a consent form before this study. The patients’ capacity to consent to participate was established with a capacity assessment, which was conducted by a registered clinical psychologist who was not part of this study.

### Participants

A total of 60 patients with dementia were screened for inclusion. A history of severe motion sickness, vertigo, or impaired vision was set as an exclusion criterion. Patients confined to bed were also excluded. Per these exclusion criteria, 47 participants were eligible for participation. A total of 27 patients with dementia were deemed capable of consenting to participate in this study, of whom 20 consented and 7 declined. Therefore, this study included 20 patients with dementia.

In total, 20 in-patients with mild to severe dementia (male: n=7; female: n=13; age: mean 73.15, SD 16.17 years) participated in this study. The diagnosis was confirmed by using the Mini-Mental State Examination (MMSE) [24]. Our participants had a mean MMSE score of 15.10 (SD 6.16), ranging from 3 to 24 (mild: n=6; MMSE score: mean 22.17, SD 1.52; moderate: n=8; MMSE score: mean 15.25, SD 1.67; severe: n=6; MMSE score: mean 7.83, SD 3.82). Participants had no prior experience of using VR. All participants had normal or corrected vision and no history of severe motion sickness.

A total of 16 health care professionals (HCPs; male: n=2; female: n=14; age: mean 27.5, SD 5.89 years) were recruited to evaluate the system’s usability and to identify the design challenges and opportunities. Their professions included caregiving or nursing (n=10), as well as occupational and speech therapy (n=6).

### Instruments

To overcome the communication difficulties associated with dementia, patients were escorted during the data collection process by their caregivers. The data were also collected by a psychologist with experience in working with patients with dementia.

#### Heart Rate

Previous research has suggested that heart rate provides a valid and reliable measure of the psychophysiology of emotions [25]. Therefore, we measured the participants’ heart rate every second to identify emotional anticipation. The heart rate was measured through optical heart rate monitoring, using photoplethysmography. This method uses light and the changes in the amount of light absorbed by the skin to measure changes in blood volume.

#### Eye-Tracking Data

The technology used in this study allowed for the analysis of the behavior and gaze patterns of patients with dementia, thus providing the opportunity to gain a better understanding of what they were experiencing. Specifically, we collected data on which objects they were looking at over time and how long they spent looking at these objects.

#### Time

Patients with dementia could spend up to 15 minutes exposed to VR. The time exposed to VR was measured in minutes and seconds. This time was measured in order to determine the patients’ interest in VR and to record the side effects.

#### Overt Aggression Scale-Modified for Neurorehabilitation

The Overt Aggression Scale-Modified for Neurorehabilitation (OAS-MNR) [26] allows for the continuous direct observation and assessment of antecedents, contexts, behaviors, and interventions. It records the type and severity of aggression, using the following four categories: verbal aggression, physical aggression against objects, physical aggression against self, and physical aggression against others. The scale was administered to the patients with dementia before, during, and after the VR session to evaluate their aggressive responses.

#### Observed Emotion Rating Scale

The Observed Emotion Rating Scale (OERS) [27] allows for the direct observation of the time spent expressing the following five affect types: pleasure, anger, anxiety, sadness, and general alertness. For patients with dementia, this time was measured in minutes and seconds (1=never; 2=less than 16 s; 3=16-59 s; 4=1-5 min; 5=more than 5 min) before, during, and after the VR session in order to assess the presence of BPSDs.

#### Visual Analog Scale

A visual analog scale (VAS) [28] was used to obtain data on the emotional reactions toward each virtual environment. The patients with dementia were asked to point to the emoji (0=happy; 5=sad) that matched their emotional state before, during, and after the VR session.

#### Slater-Usoh-Steed Questionnaire

The Slater-Usoh-Steed Questionnaire [29] assesses the level of presence and immersion through questions rated on a 7-point Likert scale (eg, 1=being somewhere else; 7=being in the virtual environment). The scale was administered after VR exposure to both the patients with dementia and the HCPs in order to assess the level of presence and immersion and to inform the design of the VR system.

#### System Usability Scale

The System Usability Scale [30] evaluates a system’s usability by using questions rated on a 5-point Likert scale (1=strongly disagree; 5=strongly agree). The scale was administered to the HCPs after the use of the VR system, to inform the design of the system.

### Study Design and Procedure

**Figure 1.**
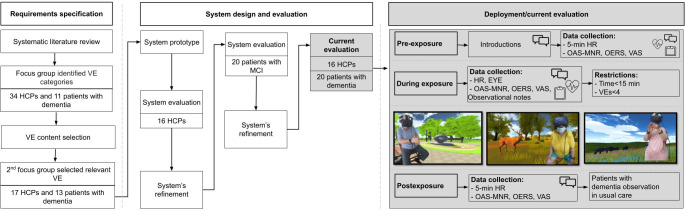
Study design and procedure. EYE: eye-tracking data; HCP: health care professional; HR: heart rate; MCI: mild cognitive impairment; OAS-MNR: Overt Aggression Scale-Modified for Neurorehabilitation; OERS: Observed Emotion Rating Scale; VAS: visual analog scale; VE: virtual environment.

The study design emerged from a systematic review that examined the feasibility of VR for people with neurological disorders and dementia, as well as discussions with experts in the field [31]. Data were collected within a 3-month period and included data on interviews, quantitative subjective responses, and physiological reactions. These data were obtained from HCPs, older adults with mild cognitive impairment (MCI), and patients with dementia (Figure 1). Specifically, first, we identified the VR system’s requirements and developed an initial prototype (further details can be found in the *Affective Experiences in VR* and *Virtual Environment Selection Process* sections). Second, we evaluated the system’s usability and sense of presence with 16 HCPs. Each HCP used the system as a user and as an administrator. Third, we refined the system based on the HCPs’ comments, and we conducted a pilot study with 20 older adults with MCI to inform the design of the system. Based on the feedback we received from the older adults with MCI, we refined the system again and re-evaluated it with the 16 HCPs. Finally, we evaluated the final product with patients with dementia. All aspects of the design process are documented and published [32].

Trials in the final evaluation started with the recording of pre-exposure measures before the VR session. This included recording the heart rate for 5 minutes, completing the VAS questionnaire, and recording responses to the OAS-MNR and OERS. Participants were then provided with an A3-sized sheet of paper presenting a “menu” with pictures of the available virtual environments to choose from. Each participant could choose up to 3 virtual environments to experience. Afterward, VR was introduced to the participants. To prevent adverse effects, such as dizziness associated with VR, a maximum duration of 15 minutes was suggested. However, during the exposure, some patients with dementia refused to remove the headset, so 5 more minutes were offered to them. During the VR session, various during-exposure measures were taken. This included recording the heart rate and eye-tracking−related metrics, filling in the VAS questionnaire after experiencing each virtual environment, and recording OAS-MNR and OERS responses and observational notes. As part of the postexposure measures, participants completed a semistructured interview and filled in quantitative data (ie, VAS, Slater-Usoh-Steed Questionnaire, and System Usability Scale questionnaires). The heart rate was also recorded for a 5-minute period after exposure. On average, each session lasted 40 minutes.

### Apparatus

The VR system for this study was developed by the authors, using the Unity3D (Unity Technologies) [33] game engine, and the 3D models were retrieved from the Unity Asset Store and repurposed to run on a VIVE Pro Eye VR system (HTC Corporation) [34]. The VR content was streamed on a laptop screen, mirroring the real-time views of patients with dementia. The gazes of patients with dementia were tracked through the head-mounted display’s eye tracker and visualized by using a ray that was only visible on the laptop screen. The ray, which was based on the direction of the gaze of the patient with dementia and their position, could indicate where the patients were looking. By casting the ray toward the virtual environment, we were able to identify which object in the virtual world patients with dementia were looking at, via collision detection with the various points of interest in the environment. Finally, a Samsung Galaxy Active 2 (Samsung Electronics) [35] smartwatch was worn by the participants and tracked their heart rate. For this purpose, a smartwatch-based app was developed by using Tizen Studio (Tizen Project), which communicated (via Bluetooth) with a paired mobile device that recorded the heart rate data every second via a mobile app that was developed by using Eclipse (IBM Corporation).

### Data Analysis

An analysis of presence and exposure time data was conducted, using descriptive statistics. Further, an analysis of system usability was performed based on ratings, which ranged from 0 to 100. The ratings were calculated by subtracting 1 from participants' responses to positive statements and subtracting 5 from participants' responses to negative statements. The resulting values were then added and multiplied by 2.5 to yield the final rating. The heart rate data followed a normal distribution; thus, a repeated measures ANOVA was performed. For the eye-tracking data, descriptive statistics were run to identify the virtual environments that were of interest to patients with dementia and determine the amount of time patients with dementia spent looking at different groups of objects within the virtual environments. Finally, frequencies were used to report on the OAS-MNR, and Friedman tests were performed to report on the VAS and the observed emotions (pleasure, anger, anxiety, sadness, and general alertness), which were compared before, during, and after VR exposure. Means and SDs were reported. For statistical tests, an α of .05 was used to test significance.

## Results

### Virtual Environment Selection Process

The virtual environments that were used in the developed system were selected by using a multistep process. First, a 2-hour workshop was conducted with 34 specialists in dementia care and 11 patients with dementia. During the workshop, attendees were asked to brainstorm the types of VR content that patients with dementia would like to receive as interventions. Attendees suggested the following categories: (1) *Travel*, (2) *Nature*, (3) *Arts Experience*, (4) *Hobbies and Sports*, (5) *Social*, (6) *Home*, (7) *Pets*, and (8) *Familiar Patient-Content*.

After the conclusion of the workshop, we systematically searched the Unity Asset Store, using the human-computer interaction Bargas-Avila and Hornbæk methodology [32], for all the relevant assets and environments. Overall, we identified 150 potentially relevant assets and environments. Exclusion criteria were then applied, which narrowed down the available content to 55 virtual environments. We excluded (1) nonpreassembled virtual environments (eg, we excluded packages that only included individual models or did not include already designed virtual environments) to provide the patients with dementia with the sense of being in the environment; (2) intimidating or scary content (eg, we excluded animals or people that were close to the user); and (3) virtual environments with a total of >1,000,000 triangles to avoid simulator sickness and long loading times.

**Figure 2.**
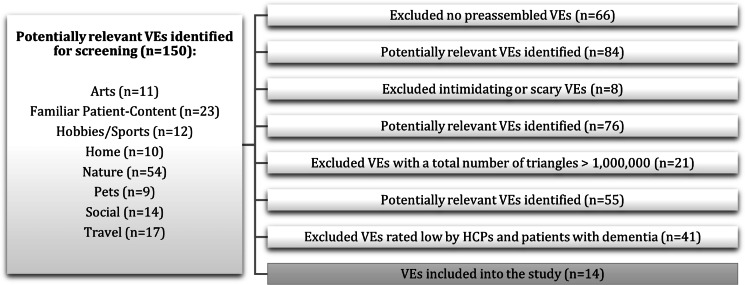
Identification and selection process for the VEs included in the virtual reality system. HCP: health care professional; VE: virtual environment.

**Figure 3.**
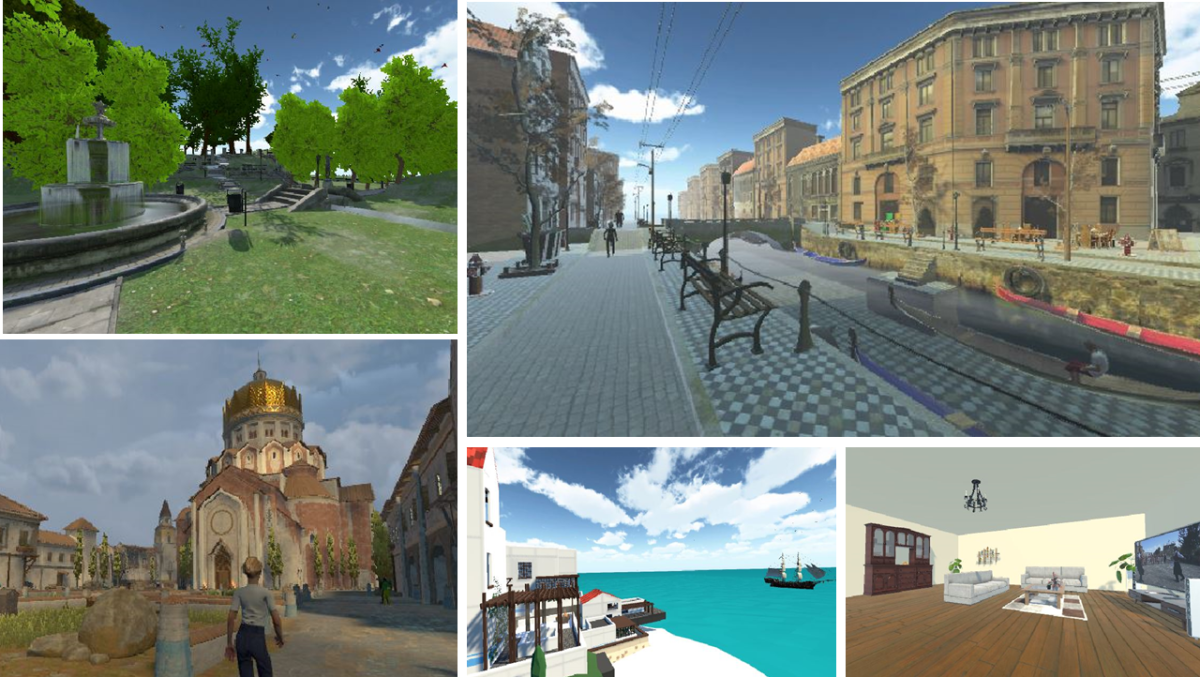
Snapshots of 5 of the 14 virtual environments that were offered to the patients with dementia.

Two focus groups with 17 specialists (group 1) and 13 patients with dementia (group 2) were then conducted to rate the available virtual environments. Through this process, 14 virtual environments were selected for final inclusion, as can be seen in Figure 2, which presents the identification and selection process. All of the virtual environments were enhanced with sounds (eg, sounds of nature and traditional music), videos (eg, traditional dancing, cooking shows, and movies), animals (eg, birds, cats, cows, and deer), people, and other elements. Snapshots of some of the virtual environments that were included in this study are displayed in Figure 3.

### System Usability and Presence

High ratings for presence (maximum score of 7) were reported by both patients with dementia (mean 6.4, SD 0.92) and HCPs (mean 4.82, SD 1.49). HCPs reported high ratings for system usability, with an average score of 71.63.

### Exposure Time

Our findings suggested that 17 of 20 patients with dementia completed the 15-minute VR session, and almost all (n=15) requested a longer exposure time; some of the participants refused to remove the headset, and up to 5 more minutes were offered to them (exposure time: mean 16.08, SD 1.63 minutes). Only 3 out of 20 patients with dementia requested to limit the exposure time due to the headset’s properties (eg, the headset was too heavy for them and blocked their normal breathing; exposure time: mean 4.79, SD 2.52 minutes). The overall average exposure time was 14.38 (SD 4.47) minutes. No adverse effects, such as motion sickness or dizziness, were reported by the patients with dementia during or after VR use.

### Heart Rate

**Figure 4.**
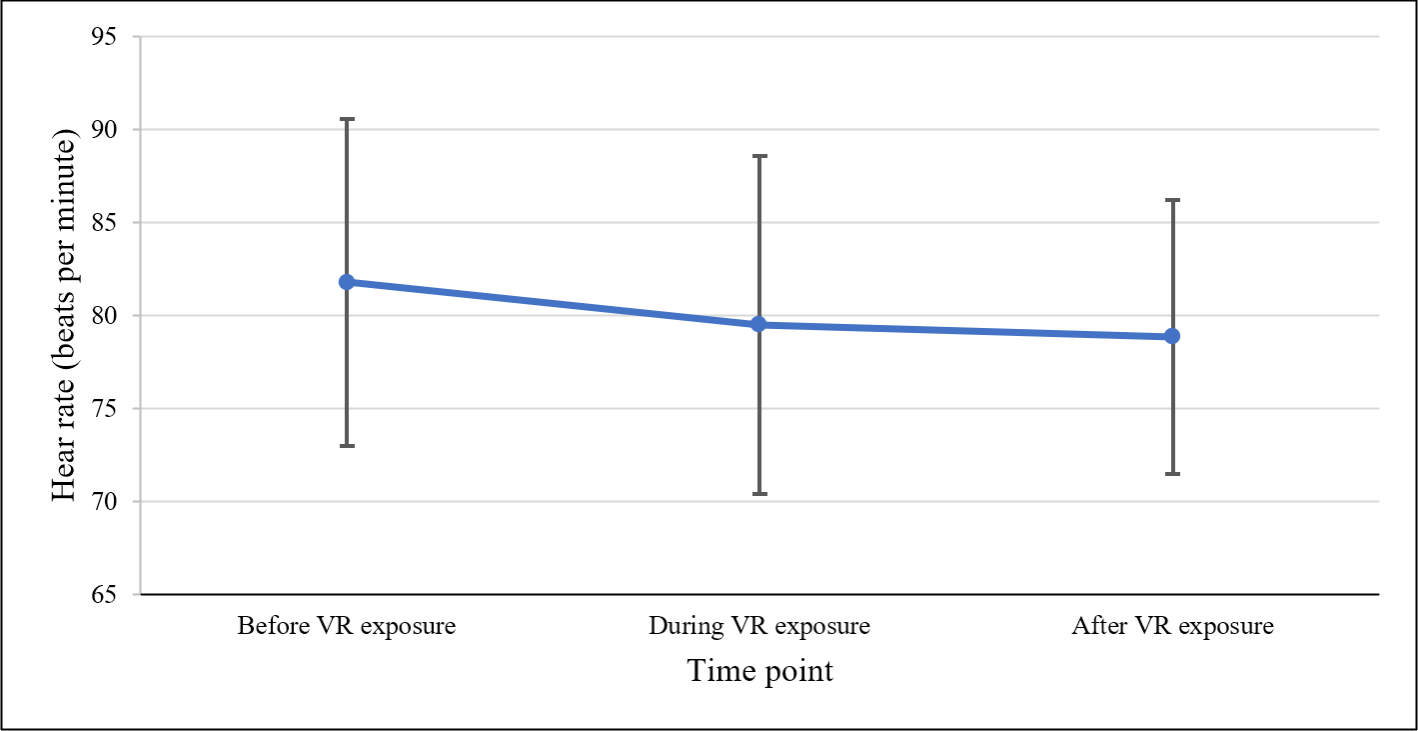
Heart rate before, during, and after VR exposure. VR: virtual reality.

The heart rate measurements of patients with dementia before, during, and after VR exposure were compared. The repeated measures ANOVA indicated that heart rates were significantly different among the three measurement time points (*F*_2,17_=5.86; *P*=.007). A post hoc pairwise comparison with Tukey correction showed a significant decrease in heart rate from before to during VR exposure (*Z*=2.76; *P*=.03) and a significant decrease from before to after VR exposure (*Z*=2.65; *P*=.04). No significant difference was found between heart rate during VR exposure and heart rate after VR exposure (*Z*=0.88; *P*=.66). These findings indicate the ability of VR to reduce the heart rates of patients with dementia (Figure 4).

**Figure 5.**
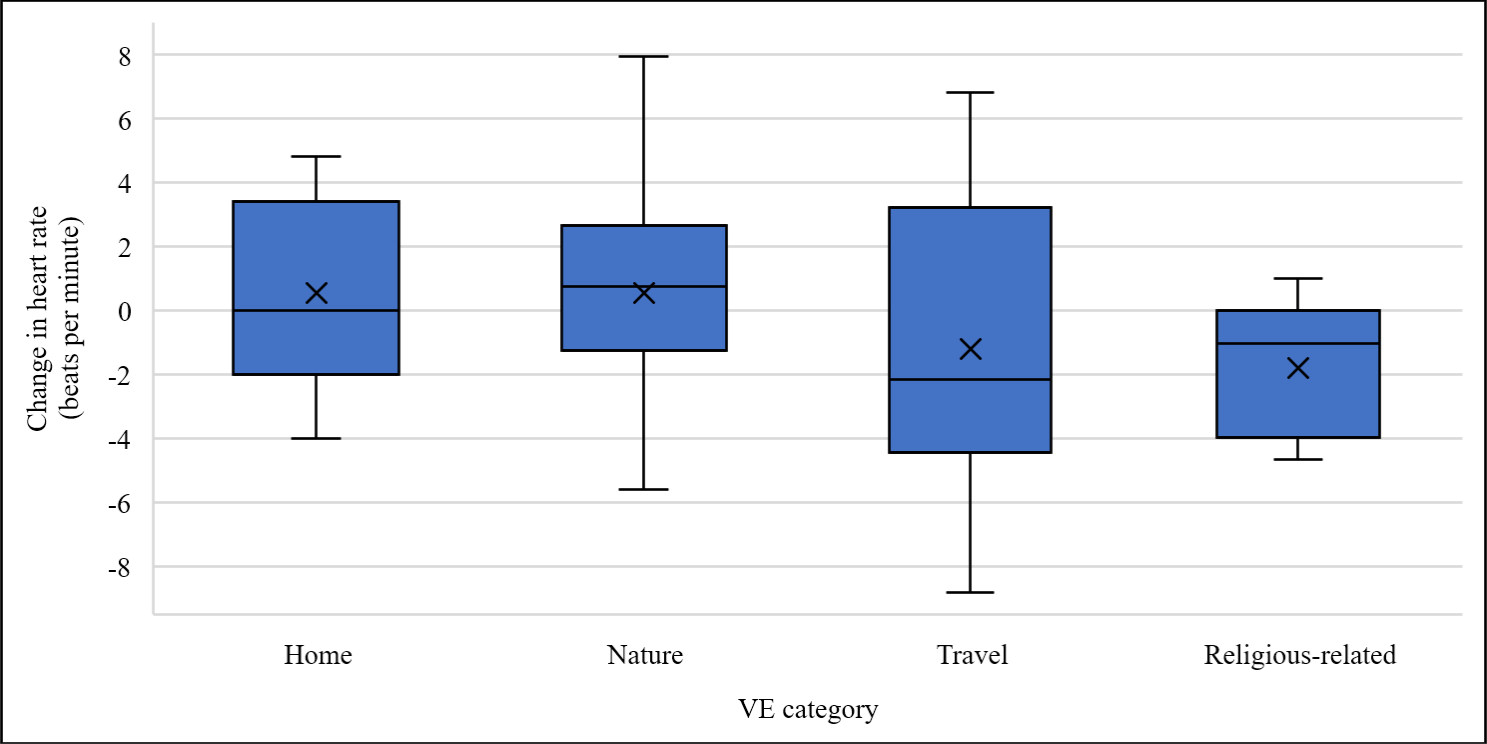
Box and whisker plot showing the change in heart rate from before virtual reality exposure to after virtual reality exposure for each thematic category of VEs. VE: virtual environment.

The 14 virtual environments were grouped into different categories based on their context (eg, nature scenes, travel destinations, etc). Figure 5 shows the change in heart rate from the start of VR exposure to the end of VR exposure for the virtual environment categories to which at least two patients with dementia were exposed. The categories that resulted in the highest decrease in heart rate measures were religious-related content (mean −1.81, SD 2.1 beats per minute) and travel (mean −1.22, SD 5.25 beats per minute).

### Eye Tracking

As previously mentioned, patients with dementia were provided with an A3-sized sheet of paper presenting a “menu” with pictures of the available virtual environments to choose from. Each patient with dementia could choose up to 3 virtual environments (n=60). Descriptive statistics indicated that patients with dementia viewed a total of 52 different environments. Most patients with dementia requested to be exposed to environments relevant to nature (30/52, 58%). This was followed by familiar places, such as home environments (10/52, 19%), environments related to traveling (6/52, 12%), and religious places (4/52, 8%). It is worth noting that during their exposure to religious places, the patients with dementia reacted as if the virtual place was a real holy place (eg, patients with dementia crossed themselves while entering the temple environment). These reactions indicate high levels of immersion and presence. Patients with dementia showed limited interest in sport-related virtual environments (2/52, 4%).

Table 1 presents the amount of time patients with dementia spent looking at different groups of objects within the virtual environments, based on the total time they were exposed to VR. Almost 33% of the time was spent looking at background elements, such as the atmosphere (eg, the sky, clouds, etc) and the ground (eg, grass and floor). Various naturalistic elements were also widely looked at (eg, 13.93% of the time was spent looking at trees, plants, etc; around 6% of the time was spent looking at water, hills, etc). Patients with dementia were also attracted to moving and active objects within the virtual environments, spending 9.22% of the time looking at them. For example, these included cars racing, boats sailing, and content playing on a television. Further, relative to their sparsity within the virtual environments, the time patients with dementia spent looking at animals was also noteworthy.

**Table 1.** The percentage of time people looked at different categories of objects within the virtual environments.

Object category	Viewing time, %^a^
Atmosphere	16.65
Ground	16.14
Flora	13.93
Motion/active objects	9.22
Water	6.52
Nature	5.97
Wall	5.65
Religious place	5.07
Building	4.56
Furniture	4.2
Animal	3.87
Item	3.1
Human	1.6
Art	1.47
Decoration	0.8
Other	1.25

a Amount of time spent looking at an object over the total time of virtual reality exposure.

### Affective Experiences in VR

A range of data sources was analyzed to identify the affects experienced by patients with dementia in VR. As can be seen from the following results, VR usage was associated with many positive emotions.

#### The OAS-MNR

Overall, 6 of 20 in-patients presented aggressive behaviors (verbal aggression: n=3; physical aggression toward self: n=3). There was a reduction in the frequency and severity of overt aggression before the VR exposure compared to those during and after the VR exposure. The aggregate aggression score was calculated by multiplying frequency by the mean weighted severity. An aggregate aggression score of 9 was calculated before VR exposure, which decreased to 0 during and after VR exposure.

#### The OERS

The Friedman test indicated that ratings of pleasure before, during, and after VR exposure significantly differed (*χ*^2^_2_=25.200; *P*<.001). Wilcoxon signed-rank tests revealed a significant increase in pleasure from before to during VR exposure (*Z*=–3.755; *P*<.001), from before to after VR exposure (*Z*=–3.140; *P*=.002), and from during to after VR exposure (*Z*=–2.683; *P*=.007). These findings suggest that patients with dementia feel pleasure and have positive emotions when they are immersed in VR. These findings also suggest a reduction in positive emotions once patients return to reality, but in comparison to the pre-exposure reports, pleasure was still significantly high.

Ratings of anger before, during, and after VR exposure significantly differed (*χ*^2^_2_=31.902; *P*<.001). Wilcoxon signed-rank tests revealed a significant decrease in anger from before to during VR exposure (*Z*=–3.873; *P*<.001) and from before to after VR exposure (*Z*=–3.637; *P*<.001). There was no significant difference between anger during VR exposure and anger after VR exposure (*P*=.40).

The Friedman test indicated that ratings of anxiety and fear before, during, and after VR exposure significantly differed (*χ*^2^_2_=25.750; *P*<.001). Wilcoxon signed-rank tests revealed a significant decrease in anxiety and fear from before to during VR exposure (*Z*=–3.579; *P*<.001) and from before to after VR exposure (*Z*=–3.453; *P*=.001). No significant differences were reported between anxiety and fear during VR exposure and those after exposure (*P*=.76).

Similarly, the ratings of sadness before, during, and after VR exposure were significantly different (*χ*^2^_2_=18.746; *P*<.001). Wilcoxon signed-rank tests revealed a significant decrease in sadness from before to during VR exposure (*Z*=–3.367; *P*=.001) and a significant increase in sadness from during to after VR exposure (*Z*=–3.015; *P*=.003). There was no significant difference between sadness before VR exposure and sadness after VR exposure (*P*=.07).

Finally, ratings of general alertness before, during, and after VR exposure significantly differed (*χ*^2^_2_=36.701; *P*<.001). Wilcoxon signed-rank tests revealed a significant increase in general alertness from before to during VR exposure (*Z*=–3.931; *P*<.001), from before to after VR exposure (*Z*=–3.834; *P*<.001), and from during to after VR exposure (*Z*=–3.517; *P*<.001). These findings suggest that patients with dementia reacted with alertness (ie, participated in the VR interactions, maintained eye contact with and followed objects like animals and persons in the virtual environment, looked around the room, turned their body to have a better view, and talked about and described the virtual environment) and had positive emotions when they were immersed in VR. These findings also suggest that alertness persisted after the VR exposure (Table 2).

**Table 2.** Observed ratings of emotions before, during, and after virtual reality (VR) exposure from the Observed Emotion Rating Scale.

Affect and VR exposure time point			Rating, mean (SD)	Rating, median	Phase	*P* value
**Pleasure**						<.001
	Before VR exposure		1.80 (0.95)	2.00	Before VR exposure to during VR exposure	<.001
	During VR exposure		3.55 (0.94)	3.00	Before VR exposure to after VR exposure	.002
	After VR exposure		2.80 (0.41)	3.00	During VR exposure to after VR exposure	.007
**Anger**						<.001
	Before VR exposure		2.35 (1.04)	2.00	Before VR exposure to during VR exposure	<.001
	During VR exposure		2.19 (0.51)	2.00	Before VR exposure to after VR exposure	<.001
	After VR exposure		1.28 (0.33)	1.25	During VR exposure to after VR exposure	.40
**Anxiety and fear**						<.001
	Before VR exposure		2.65 (2.00)	1.23	Before VR exposure to during VR exposure	<.001
	During VR exposure		1.25 (1.00)	0.44	Before VR exposure to after VR exposure	.001
	After VR exposure		1.20 (1.00)	0.52	During VR exposure to after VR exposure	.76
**Sadness**						<.001
	Before VR exposure		2.40 (1.09)	2.00	Before VR exposure to during VR exposure	.001
	During VR exposure		1.23 (0.38)	1.00	Before VR exposure to after VR exposure	.07
	After VR exposure		1.77 (0.41)	1.71	During VR exposure to after VR exposure	.003
**Alertness**						<.001
	Before VR exposure		1.35 (0.49)	1.00	Before VR exposure to during VR exposure	<.001
	During VR exposure		4.03 (0.74)	4.00	Before VR exposure to after VR exposure	<.001
	After VR exposure		3.04 (0.90)	3.00	During VR exposure to after VR exposure	<.001

#### The VAS

**Figure 6.**
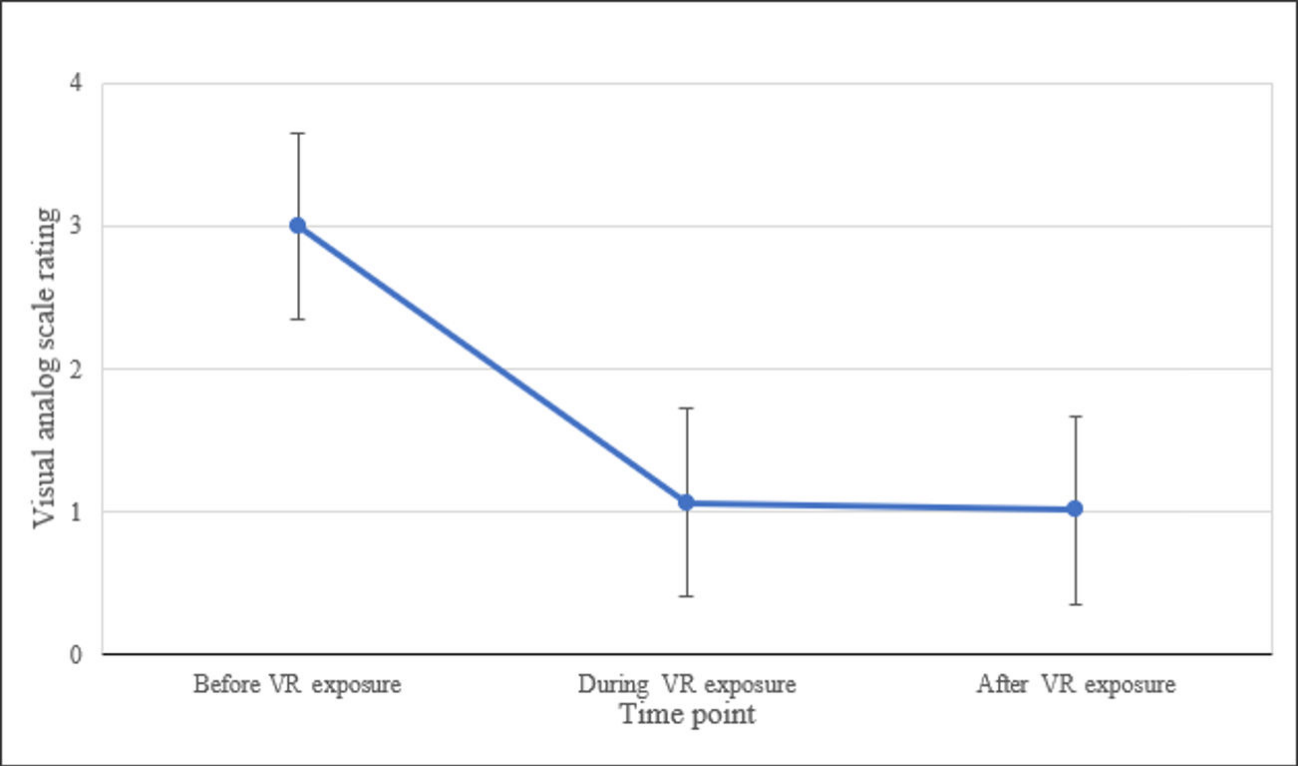
Emotions before, during, and after VR exposure (measured using a visual analog scale). VR: virtual reality.

The Friedman test indicated that the negative emotions before, during, and after VR exposure were significantly different (*χ*^2^_2_=31.66; *P*<.001). Wilcoxon signed-rank tests revealed a significant decrease in negative emotions (and a significant increase in positive emotions) from before to during VR exposure (*Z*=–3.735; *P*<.001) and from before to after VR exposure (*Z*=–3.836; *P*<.001). However, there was no significant difference between the emotional state of the person during the VR exposure and that after the VR exposure (*P*=.64; Table 3, Figure 6).

**Table 3.** Emotions before, during, and after virtual reality (VR) exposure (measured using a visual analog scale).

		Rating, mean (SD)	Rating, median	Phase	*P* value
**Emotional state**					<.001
	Before VR exposure	3.00 (1.45)	2.50	Before VR exposure to during VR exposure	<.001
	During VR exposure	1.07 (1.19)	0.83	Before VR exposure to after VR exposure	<.001
	After VR exposure	1.01 (1.22)	0.83	During VR exposure to after VR exposure	.64

## Discussion

### Principal Findings and Implications for Design

The importance of supporting the health-related quality of life of patients with dementia, including mental health and well-being, is undoubted. Current research suggests that VR can be a reliable, feasible, and acceptable solution that can promote engagement and provide an enjoyable experience for patients with dementia [18,19]. This paper describes how 24 patients with dementia, 20 patients with MCI, and 51 medical experts co-designed a VR system for reducing BPSDs among patients with dementia residing in long-term care. This paper also presents the evaluation of the system, which was carried out with 20 patients with dementia and 16 medical experts. Our findings suggest that VR encompasses several therapeutic benefits for patients with dementia. Particularly, it was shown that VR can be very effective for the reduction of BPSDs and, especially, the reduction of aggressive, agitated, anxious, apathetic, depressive, and fearful behaviors.

The results validated that VR could result in a significant improvement in BPSDs, which are highly associated with poor well-being for patients with dementia residing in long-term care [36-38]. As we have shown in the *Results* section, the recording and analysis of physiological data allow for a better understanding of the emotional states of patients with dementia during VR exposure. Earlier research has shown that stress can affect heart rate [39]; therefore, particularly promising results from our study were the heart rate data collected before, during, and after VR session. The analysis revealed a significant decrease in heart rate from before to during (*P*=.03) and after (*P*=.04) VR exposure, validating that VR can significantly reduce stress levels for patients with dementia. Therefore, these findings are especially important, since they triangulate the validation of the effectiveness of VR for patients with dementia.

To reduce BPSDs, exposure to outworld and low-stimulus experiences have been suggested in the general literature [40-42]. We support this suggestion and recommend that VR environments incorporate out-of-reach experiences that are enhanced by animals, artistic content, natural environments, and travel destinations.

In particular, we found that animals, such as cows, donkeys, birds, and cats, among others, can benefit patients with dementia, and this is in line with previous research suggesting that watching animal content can reduce cardiovascular responses, stress, and anxiety and can generally benefit the health of patients with dementia [43-45].

We also included music and dancing festivals as part of our VR content. We found that such content, within VR, can replicate the findings of the existing literature, as it can create meaningful experiences, reduce stress and anxiety [46,47], and increase communication between caregivers and patients with dementia [48]. Similarly, consistent with previous studies, we found that patients with dementia reminisced when they were exposed to environments with which they were familiar [49]. This is an important finding, since reminiscence therapy is recommended as a person-centered approach for treating dementia [50]. Even though the system was not fully personalized, we managed to use familiar bodies (eg, traditional dancing festivals and older-style homes) that closely matched the memories of patients with dementia. Therefore, to successfully design a VR system for patients with dementia, the use of elements that patients are familiar with is essential, since such elements trigger memories of their past.

Per our findings, we also advise adding naturalistic environments. Based on previous research, nature viewing can enhance emotional well-being and aid recovery from stress [51,52]. Indeed, nature-related environments were the most commonly chosen virtual environments among our patients with dementia, and based on our findings, nature was able to enhance emotional well-being and aid recovery from stress. 53,54

### Conclusions, Limitations, and Future Directions

This paper describes a study that examines the design and development of a VR system for patients with dementia. In contrast to prior works, to overcome limitations in the current literature, the system was co-designed with a total of 24 patients with dementia, 20 patients with MCI, and 51 experts in dementia care and was evaluated with a larger population (compared to other relevant studies) of patients with mild to severe cases of dementia. Based on our findings, VR can enhance the health-related quality of life of patients with dementia, as it encompasses a wide range of therapeutic benefits. VR was shown to be especially effective in reducing heart rate and aggressive, agitated, anxious, depressive, and fearful behaviors associated with BPSDs.

A major limitation of this study is that we evaluated the system only in a single trial. In the future, it will be necessary to conduct a longitudinal study to determine whether the positive results are sustainable and determine the clear benefits of the permanent deployment of VR in health care. Another limitation of this study is that the focus was exclusively on evaluating the VR solution in health facilities, and help was provided to the patients when administering the VR system. In recent years, low-cost, immersive VR consumer systems have been developed and released. Therefore, we suggest that future studies evaluate the use of an affordable, home-based VR solution for patients with dementia. Additionally, while this study provides the basis for conducting the first trial to evaluate the effect of VR therapy on BPSDs validated by physiological responses (eg, heart rate and eye tracking) in care hospitals, a limitation of this study is that it did not correlate the heart rate data with the gaze data. We, therefore, suggest that future studies correlate heart rate with what patients with dementia are looking at within the virtual environment and use this information for the creation of more personalized experiences. Further research is also warranted on the analysis of the gaze behaviors of patients with dementia for diagnostic purposes and for understanding their affective states during VR exposure. Ultimately, we hope that the results from this study will offer insight into how VR technology can be designed, deployed, and used in dementia care.

## Abbreviations

BPSD: behavioral and psychological symptom
HCP: health care professional
MCI: mild cognitive impairment
MMSE: Mini-Mental State Examination
OAS-MNR: Overt Aggression Scale-Modified for Neurorehabilitation
OERS: Observed Emotion Rating Scale
VAS: visual analog scale
VR: virtual reality
